# Mechanisms of Zika astrocyte infection and neuronal toxicity

**DOI:** 10.1515/nipt-2022-0014

**Published:** 2023-03-25

**Authors:** Courtney Veilleux, Eliseo A. Eugenin

**Affiliations:** Public Health Research Institute (PHRI), New York, USA; Deparment of Microbiology, Biochemistry, and Molecular Genetics, Rutgers New Jersey Medical School, Rutgers the State University of New Jersey, Newark, NJ, USA; Department of Neurobiology, University of Texas Medical Branch (UTMB), Galveston, TX, USA

**Keywords:** autophagy, brain, development, flaviviruses, microcephaly, mosquito, neuronal

## Abstract

**Objectives:**

Zika virus (ZIKV) has become an epidemic in several countries and was declared a major public health issue by the WHO. Although ZIKV infection is asymptomatic or shows mild fever-related symptoms in most people, the virus can be transmitted from a pregnant mother to the fetus, resulting in severe brain developmental abnormalities, including microcephaly. Multiple groups have identified developmental neuronal and neuronal progenitor compromise during ZIKV infection within the fetal brain, but little is known about whether ZIKV could infect human astrocytes and its effect on the developing brain. Thus, our objective was to determine astrocyte ZiKV infection in a developmental-dependent manner.

**Methods:**

We analyze infection of pure cultures of astrocytes and mixed cultures of neurons and astrocytes in response to ZIKV using plaque assays, confocal, and electron microscopy to identify infectivity, ZIKV accumulation and intracellular distribution as well as apoptosis and interorganelle dysfunction.

**Results:**

Here, we demonstrated that ZIKV enters, infects, replicates, and accumulates in large quantities in human fetal astrocytes in a developmental-dependent manner. Astrocyte infection and intracellular viral accumulation resulted in neuronal apoptosis, and we propose astrocytes are a ZIKV reservoir during brain development.

**Conclusions:**

Our data identify astrocytes in different stages of development as major contributors to the devastating effects of ZIKV in the developing brain.

## Introduction

Zika virus (ZIKV) was declared a Public Health Emergency by the World Health Organization (W.H.O.) from February 2016 to March 2017 [1, 2]; however, the emergence of new viruses such as SARS-CoV-2 has reduced the interest in ZIKV despite its devastating consequences during developmental stages [3], [4], [5], [6], [7]. Despite the multiple outbreaks and epidemic proportions of ZIKV worldwide, the mechanism by which this virus compromises the developing brain only recently has been explored [8], [9], [10], [11], [12], [13], [14]. ZIKV was first identified in 1952 during surveillance for the yellow fever virus in Uganda [15, 16]. It is traditionally characterized by asymptomatic infection or mild febrile disease, with recovery and protective immunity among populations surrounding forests with jungle-related transmission [17]. Reports of ZIKV-related illness before 2000 were extremely rare [18, 19]. Since 2000, the symptomatic infection has been documented in an outbreak in Micronesia in 2007 [20, 21] and an outbreak in French Polynesia from 2013 to 2015 [22, 23]. In 2015, Brazilian health officials reported the first cases of ZIKV infection [24] and a nearly 20-fold increase of microcephaly in regions heavily infected with ZIKV was identified, with rates increasing from 5.7 to 99.7 per 100,000 cases in one year, but every year new data and long term consequences are identified [25], [26], [27], [28], [29], [30].

ZIKV causes neurological and endocrine defects in the fetus during development resulting in Zika Congenital Syndrome [31]. Postmortem studies have identified Zika proteins and mRNA in the amniotic fluid of microcephalic fetuses after symptomatic maternal infection [32], as well as in the brain tissue of fetuses that developed microcephaly, ventriculomegaly and cortical mass reduction after symptomatic maternal infection [33]. Febrile illness in the mother is not required for fetal exposure and infection – nonapparent maternal infection resulted in fetal demise with multiple areas of the brain infected [34]. Recent advances in animal models have confirmed many of these findings, including the use of rodents, macaques, ferrets, and organoids [35], [36], [37], [38], [39], [40], [41], [42], [43], [44], [45]. Most published data support that ZIKV infection compromises the fetus early during development, and there are specific developmental windows that are essential for the pathogenesis of the virus, but these windows are poorly known.

*In vitro* studies have substantiated the tropism of fetal CNS tissue through the identification of ZIKV viral RNA in neurons [46], neural progenitor cells [47], [48], [49], and glial cells [50]. ZIKV-positive neurons and neural progenitor cells undergo apoptosis and/or shows dysfunction in proliferation during infection [46], mechanisms characteristic of lissencephaly and microcephaly – both of which have been identified as significant characteristics for ZIKV congenital syndrome. However, glial cells (radial glial cells and astrocytes) have also been identified to support ZIKV replication by AXL, TYRO3 and CD209 mechanisms [49]. Interestingly, ZIKV pathogenesis in the fetus is different than the time of infection in the mother due that replicating virus can be detected in the fetus after ZIKV clearance from maternal circulation [51]. Long-term neuronal apoptosis after mother infection clearance indicates that ZIKV remains in a CNS cell throughout gestation [51]. *In vitro* ZIKV infection of Neuronal Progenitor Cells (NPCs) maintain productive infection over 28 days without induction of chemokine of inflammatory signaling [48]; however, other reports demonstrated that transient infection of NPCs results in a decreased proliferation and increased cell death [52]. These data indicate that ZIKV infection and replication rely on a CNS resident cell in the developing brain. We hypothesize that undifferentiated astrocytes are one of the main ZIKV-infected cells and potential reservoirs for long-lasting ZIKV infection as well as bystander neuronal toxicity. This report used human astrocytes at different post-conception times (16–22 weeks) to examine infectivity, replication, and neuronal toxicity. Although others have identified neurons and neural progenitor cells as major ZIKV targets, few have characterized ZIKV infection in astrocytes [53], [54], [55], [56], [57], [58], [59], despite the key role of astrocytes in neuronal development and their abundance within the fetal and adult CNS. Our data demonstrate that ZIKV enters, infects, and replicates in human astrocytes in a developmental-dependent manner. The ZIKV infection of astrocytes contributes to glial and neurotoxicity by becoming a long-term “viral powerhouses” that resists apoptosis and contributes to bystander toxicity within the developing brain.

## Results

### Zika virus productively infects primary human astrocytes

**Figure 1:**
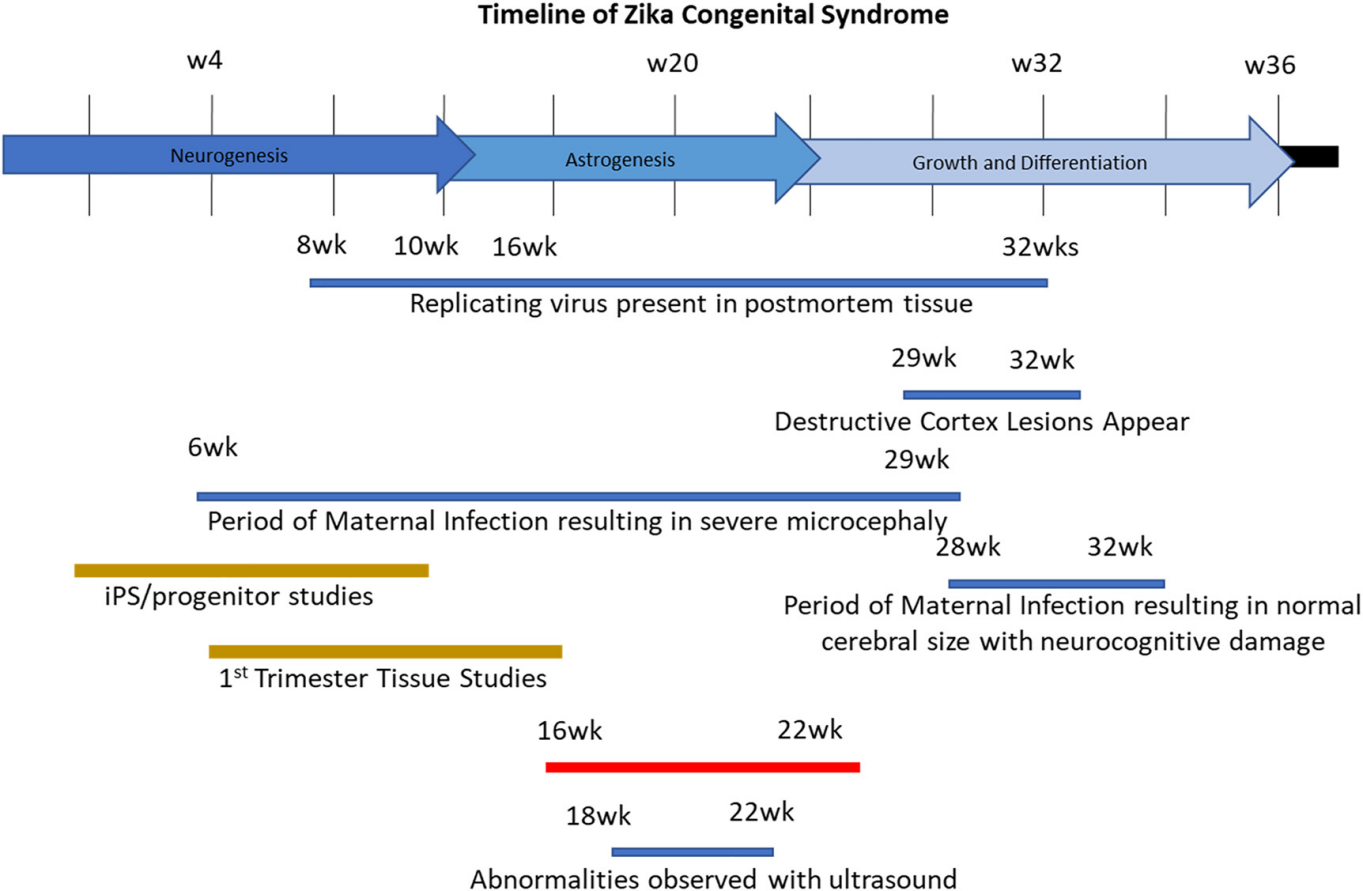
Time course of events described in ZIKV infection in humans. A summary of the published data indicating the identification of replicating virus (8–32 wpc), destructive cortex lesions (29–32 wpc), maternal infection resulting in microcephaly (6–29 wpc), and maternal infection resulting in normal brain development (blue lines, 28–32 wpc). Also, we denote the Ips and first trimester studies (brown lines) and the developmental window of our studies in correlation with the detection of brain abnormalities observed by ultrasound (red and blue lines, 16–22 and 18–22 wpc).

To characterize the sensitivity of human astrocytes to ZIKV infection during different developmental periods, we used pure astrocytes cultures or mixed cultures of human neurons and astrocytes obtained at different developmental stages to determine their infectivity and viral replication. Figure 1 represents a time course summary of ZIKV congenital syndrome as described [4, 29, 45, 53]. We denote the timeline of gestation (arrows), neurogenesis, astrogenesis, growth and differentiation, and the correlation of these neurodevelopmental stages with ZIKV infection and brain abnormalities detected by ultrasound and our studies between 16 and 22 weeks post-conception, wpc (Figure 1).

Our studies used human primary astrocytes isolated from different gestational ages (16–22 wpc) to cover the critical timeline of ZIKV infection and associated brain abnormalities observed in fetuses by ultrasound (Figure 1, bottom of the figure) [60]. Astrocyte cultures obtained at different developmental periods were exposed to ZIKV at different MOI to examine infectivity (MOI, 0.1 and 1), replication and associated toxicity.

**Figure 2:**
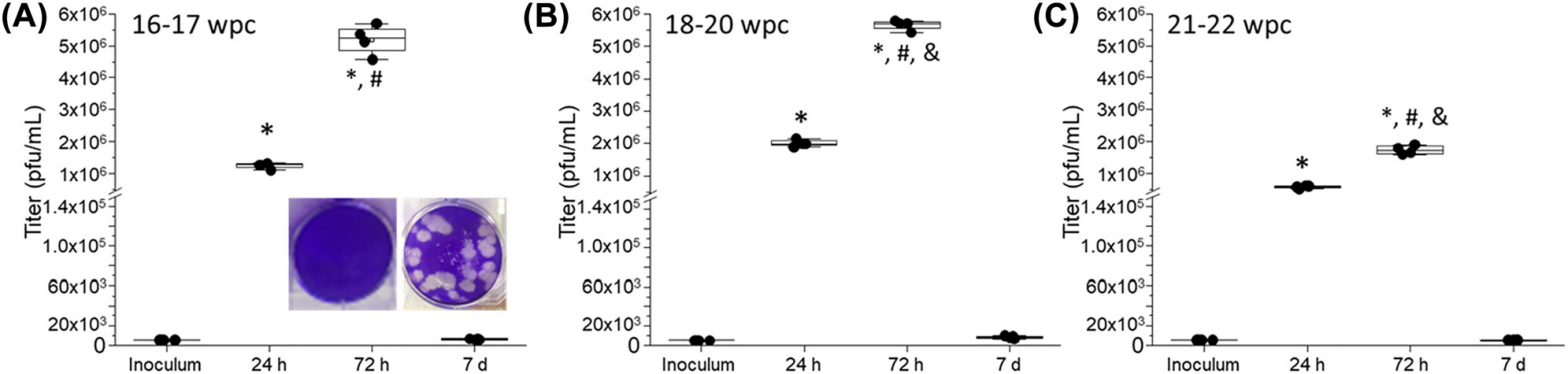
ZIKV infection of human fetal astrocytes is productive and dependent on the degree of differentiation. (A) Plaque assay to determine ZIKV secretion from human primary astrocytes, 16–17 wpc. We quantified infection after 0 (inoculum), 24 h, 72 h and 7 days post-exposure. Vero cells were inoculated with astrocyte supernatants for 7 days before fixation and plaque visualization using crystal violet. The total number of plaques was counted, and titer (pfu/mL) was calculated. Plaque formation was distinct, with large plaques dispersed throughout the monolayer. Insets indicate a mock and a ZIVK infected plate (*p≤0.001 compared to Inoculum and #p≤0.001 compared to 24 h, n=4 independent donors in quadruplicate). (B) Plaque assay to determine ZIKV secretion from human primary astrocytes, 18–20 wpc. A similar approach to A was used. Infection of astrocytes at this developmental stage was higher, similar to at early stages (*p≤0.001 compared to Inoculum, #p≤0.005 compared to 24 h, &p≤0.05 compared to 72 h in A, n=4 independent donors in quadruplicate). (C) Plaque assay to determine ZIKV secretion from human primary astrocytes, 21–22 wpc. A similar approach to A was used. Infection of astrocytes decreased compared to early stages of differentiation (*p≤0.001 compared to Inoculum, #p≤0.005 compared to 24 h, &p≤0.05 compared to 72 h in A or B, n=4 independent donors in quadruplicate). All time points analyzed resulted in higher titers than the original inoculum.

For these experiments, we decided to use an Asian ZIKV lineage due to its isolation being directly from an infected person and passaged minimally in mosquito epithelial cells (C636). This approach differs from most African ZIKV strains, including MR766, which are historically passaged in fetal mouse brains and likely have adapted to CNS tissue over time [15]. Overall, ZIKV is a member of *Flaviviridae* and produces both infectious and noninfectious virions. Subviral particles are common during Flavivirus infection and result in uptake, but not viral production, of new virions in host cells [61], [62], [63], [64], [65], [66]. To determine the ZIKV infectivity of human astrocytes at different fetal developmental stages were inoculated with ZIKV strain PA 259,459, an Asian lineage strain isolated from an infected human, Panama in 2015. Human fetal astrocytes were infected with MOI 0.1–6 × 10^5^ plaque forming units (pfu)/mL onto 500,000 cells per well— for 1 h, washed with PBS, and incubated with growth medium over a time course of 7 days to later perform plaque assays using the cell supernatants at 2 h, 72 h or 7 days post-infection (Figure 2). Plaques were counted, and the infectious titer was calculated into pfu/mL. There was no plaque formation using supernatants from mock or uninfected C636 cells (Figure 2A, Inoculum, see inset pictures for control and ZIKV-infected plaques). Inoculation with ZIKV of astrocytes cultures always resulted in higher infectious titers compared to the initial inoculum supporting active replication (Figure 2). ZIKV infection of human primary astrocytes obtained at the developmental stages, 16–17 wpc, shows a strong infection at 24 and 72 h post-ZIKV exposure to later decline after 7 days post-exposure indicating that production of infectious virus is acute (Figure 2A, *p≤0.001 compared to Inoculum, n=4 independent donors in quadruplicate). Moreover, infection peaked after 72 h post-exposure to later decay (Figure 2A, #p≤0.001 compared to 24 h, n=4 independent donors in quadruplicate). Analysis of ZIKV-exposed astrocytes obtained from an 18–20 wpc showed a similar early viral production, 24 h, compared to astrocytes from 16–17 wpc exposed to ZIKV (Figure 2B, *p≤0.001 compared to Inoculum, n=4 independent donors in quadruplicate). However, viral production at 72 h in astrocytes obtained from 18–10 wpc was significantly higher than in astrocytes obtained from 16–17 wpc (Figure 2B, *p≤0.001 compared to Inoculum, #p≤0.005 compared to 24 h, &p≤0.05 compared to 72 h in A, n=4 independent donors in quadruplicate). After 7 days, 18–20 wpc viral production decreased to almost inoculum levels (Figure 2B). Analysis of astrocyte cultures obtained from 21-22 wpc and exposed to ZIKV showed lower viral production compared to astrocyte cultures obtained from 16–17 and 18–10 wpc (Figure 2C, *p≤0.001 compared to Inoculum, #p≤0.005 compared to 24 h, &p≤0.05 compared to 72 h in A or B, n=4 independent donors in quadruplicate). These data indicate that astrocytes at early developmental stages are more susceptible to ZIKV infection than in the later stages of differentiation.

### NS1 protein production and stability depend on the astrocyte differentiation stage

**Figure 3:**
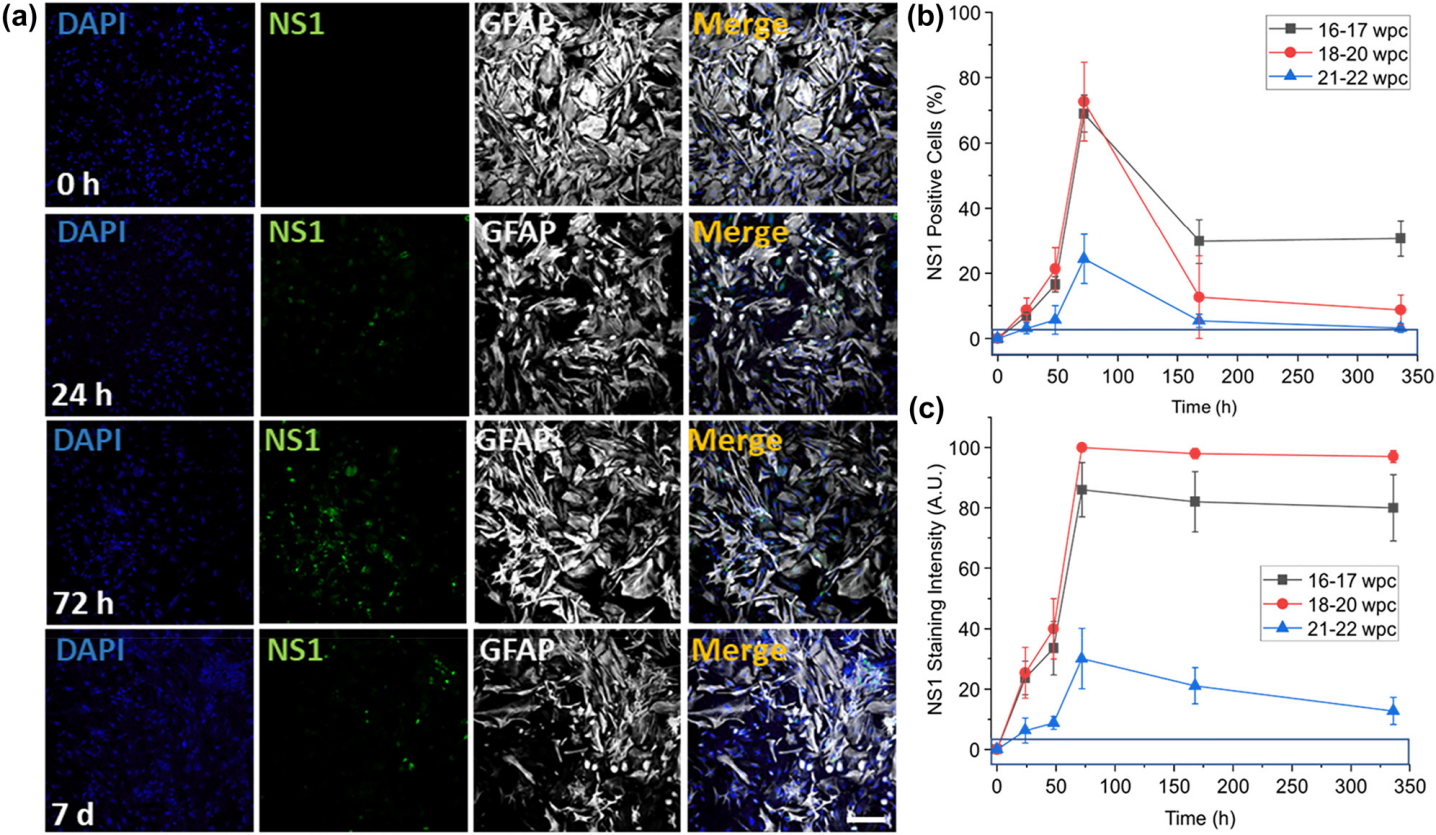
Time course of ZIKV infection in primary astrocytes and expression of the NS1 protein. Human fetal astrocytes were infected with MOI 0.1 or 1.0 ZIKV for 0, 24, 48, 72, 168 (7 days) and 336 (14 days) h. Immunostaining of the cultures for nuclei (DAPI, blue staining), NS1 (green staining), and glial cells (white staining) and subsequent confocal analysis. (A) Representative confocal images of cultures of astrocytes, 18–20 wpc, showing the increasing numbers of astrocytes positive for NS1 (24 h, 72 h, and 7 d). Bar 100 µm. NS1 staining was detected at 0 h bar: 10 µm. (B) Quantification of the percentage of astrocytes with intracellular NS1 stores. Time course of infection using astrocyte cultures derived from brains, 16–17, 18–20, and 21–22 wpc. All numbers are significant compared to the mock blue rectangle. p≤0.001 compared to Mock, n=4 independent donors in quadruplicate. (C) Quantification of the positive pixels in the astrocyte cultures described in B. All numbers for B and C were significant over the blue boxes (p≤0.001 compared to Mock, n=4 independent donors in quadruplicate).

To determine the degree of infectivity and viral protein production, we examined the time course of ZIKV infection, the number of infected astrocytes and the expression levels of NS1 protein by confocal microscopy using astrocytes from different developmental stages (Figure 3A). The time course analyzed included 0, 24, 48, 72, 168 (7 days) and 336 (14 days) h post ZIKV exposure. Immunostaining of the uninfected and ZIKV-infected cultures for nuclei (DAPI, blue staining), NS1 protein (green staining), and glial cells (white staining) and subsequent confocal analysis indicates strong NS1 positivity in astrocytes after 24–72 h (Figure 3A). ZIKV NS1 staining was accumulated in vesicle-like structures in GFAP positive cells (Figure 3A, 24 h, 72 h and 7 d). Quantification of the GFAP -positive cells with NS1 protein staining indicates that all astrocyte cultures had an intracellular accumulation of NS1 protein (16–17, 18–20, and 21–22 wpc had intracellular stores of NS1 protein); however, the degree of infectivity or staining was highly dependent on the neurodevelopmental age where the astrocyte cultures were obtained (Figure 3B and C). Astrocyte cultures obtained from older developmental stages (21–22 wpc) showed lower numbers of astrocytes containing NS1 protein (Figure 3B, all numbers are significant compared to the mock, blue rectangle. p≤0.0012 compared to Mock, n=4 independent donors in quadruplicate). Moreover, the quantification of positive pixels for NS1 indicated that NS1 staining remained constant until 14 days post-infection, suggesting NS1 intracellular protein stores had higher stability than the secreted virus (Figure 3C, p≤0.001 compared to Mock, n=4 independent donors in quadruplicate). Overall, our data indicate that NS1 expression and infection spread depend on the degree of astrocyte differentiation.

### ZIKV NS1 accumulated in enlarged vesicles within astrocytes

**Figure 4:**
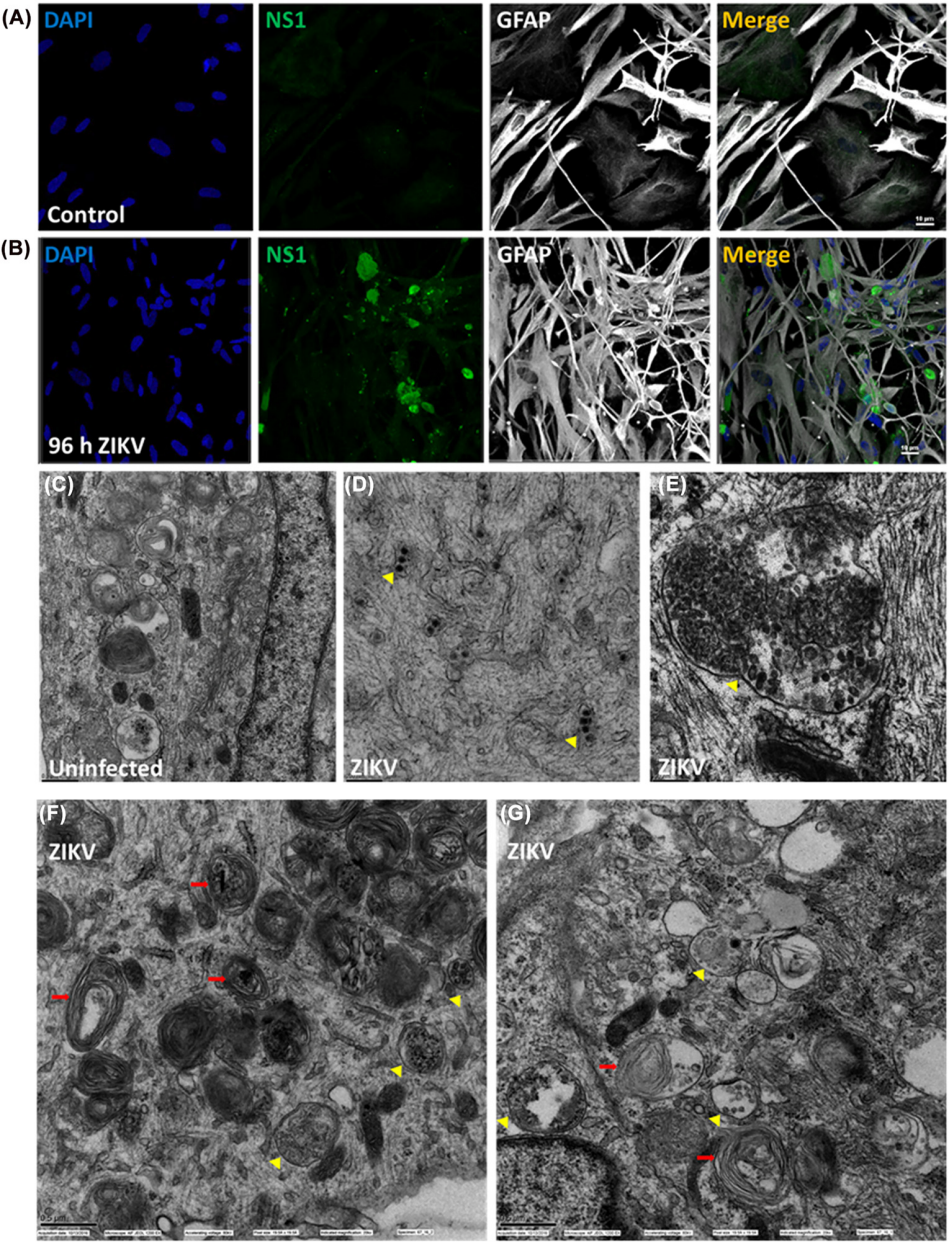
ZIKV aggregates in large vesicles in human astrocytes. Immunostaining or transmission electron microscopy of the cultures for nuclei (DAPI, blue staining), NS1 (green staining), and glial cells (white staining). (A) Mock-infected control cultures show no ZIKV-specific NS1 staining and do not cluster similarly to infected cells. (B) Fetal tissue-derived astrocytes were infected with ZIKV MOI 0.1/0.25/1.0 for 96hpi to determine the localization of the NS1 protein. ZIKV NS1 staining was localized in large vesicles in a time-dependent manner, as indicated in Figure 3. Vacuole-like structures are present within GFAP + cells. Bar: 10 µm. (C) TEM of mock-treated astrocyte cultures denoting no viral particles and exquisite ER, Golgi and vesicular structures. (D) ZIKV infected astrocyte cultures for 24–48 h. Arrows denote the presence of viral particles and a significant compromise of organelles and their interactions early after infection. (E) Human fetal astrocytes infected with MOI 0.1/1.0 for 7 days result in a total collapse of the endoplasmic reticulum and accumulation of virions within the cytoplasm. Upon closer examination, viruses appear clustered in vacuole-like structures that are not continuous vesicles and contain membrane portions throughout (yellow arrow). Bar: 0.5 µm. (F and G) Multilaminar formation into ZIKV-infected astrocyte cultures is widespread throughout the cell cytoplasm and found in proximity to autophagosome structures (red). To assess vesicle structures and organization during ZIKV infection in the developing brain, human fetal astrocytes were infected with MOI 0.5/1.0 ZIKV for 7–14 days before fixation and electron microscopy acquisition. Multilaminar structures are identifiable by their concentric loops and no distinct core. Autophagosome formation is characterized by a central core and a double membrane outer structure. Both vesicle structures were present in infected cultures and appeared scattered throughout the cell cytoplasm. Disorganized membrane structures, including ER, are also discernable by electron micrographs (Blue). Scale bar: 0.5 µm.

To determine the intracellular distribution of the virus, we performed confocal microscopy and transmission electron microscopy, TEM (Figure 4). Astrocyte culture staining for DAPI (nuclei, blue staining), NS1 protein (green staining), GFAP (an astrocyte marker), and the merge of all colors are represented in Figure 4 A and B, in uninfected control and 96 h post-ZIKV infection, respectively (Figure 4). We selected 72 and 96 h post-ZIKV infection due to the formation of large vesicles containing NS1 protein (see Figures 3 and 4A and B). No staining was detected in mock-treated or untreated cells (Figure 4A). TEM analyses of uninfected or mock-treated astrocytes cultures denote a remarkable nucleus, Golgi’s apparatus, ribosome, mitochondria, autophagosomes, and endoplasmic reticulum (Figure 4C). No detection of viral particles or like-viral particles was detected after mock or untreated conditions (Figure 4C, uninfected). To determine the time course of large vesicle formation containing NS1 as observed by confocal, TEM was performed at different time points, including 24, 48, 72, 168 (7 days) and 336 (14 days) h post ZIKV exposure (Figure 4D and E). TEM analysis indicates the early formation of viral structures (Figure 4D, 24–72 h) after ZIKV infection (Figure 4D, arrows represent viral particles). Also, a significant compromise of intracellular structures was observed, including compromised ER, Golgi, vesicles and the cytoskeleton (Figure 4D). After 72 h, a large accumulation of viral particles was observed (Figure 4E, arrows). Most virus accumulates in enlarged vesicles as indicated in the confocal analyses (Figure 4C–E, correspond to the same magnification, bar: 0.5 µm), denoting the enlarged size of the vesicles containing the virus. Also, structural changes in the ER, Golgi, and cytoskeleton remain more pronounced (Figure 4E, see red arrows). Higher magnification of the areas analyzed shows the accelerated formation of autophagosomes with their characteristic concentric membranes with a lack of distinct core or cellular material (Figure 4F and G, red arrows). We identified early stages (Figure 4D, yellow arrows) and late stages of viral accumulation in large vesicles (Figure 4E–G, yellow arrows). However, we did not find significant differences in viral accumulation or organelle dysfunction among the three types of astrocytes, 16–17/18–20 or 21–22 wpc (data not represented). Our TEM data corroborate a close relationship between the plaque assays and confocal data as well as denote significant organelle dysfunction and NS1 accumulation. NS1 accumulation has been reported for several Flaviviruses but without the developmental component and organelle dysfunction [53, 65, 67]. Overall, our data indicate that ZIKV infection, in addition to infected astrocytes in a neurodevelopmental-dependent manner, also compromises the astrocyte organelle and their interactions.

### ZIKV infection of astrocytes triggers neuronal apoptosis in an age-developmental-dependent manner

**Figure 5:**
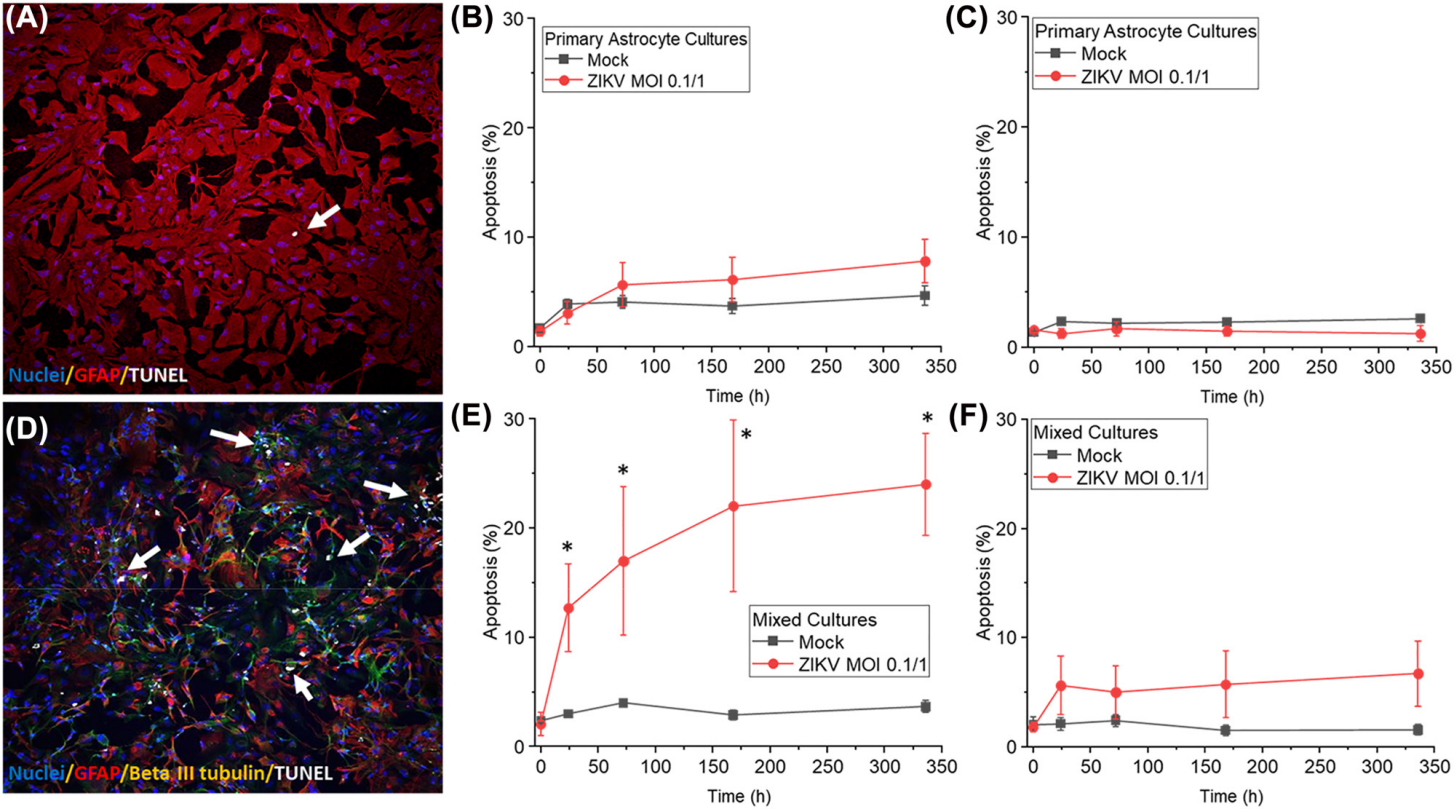
ZIKV infection protects infected astrocytes from apoptosis but increases neuronal apoptosis in a developmental-dependent manner. The human brain was dissociated and cultured into pure populations of fetal astrocytes or mixed cultures of neurons and astrocytes. Confocal analysis was completed, and the percentage of TUNEL-positive nuclei per culture was calculated in glial and neuronal cells upon staining for GFAP or neuronal tubulin III. (A) Representative image of an astrocyte culture with minimal apoptosis. (B) Quantification of apoptosis in GFAP positive cells using pure cultures of astrocytes, 16–17/18-20 wpc (data was combined due that were not significant comparing both). No significant apoptosis was detected in the ZIKV-infected cultures compared to mock infection. (C) Quantification of apoptosis using astrocyte cultures 21–22 wpc. No significant apoptosis was detected in the ZIKV-infected cultures compared to mock infection. (D) Representative confocal image of mixed cultures of neurons and astrocytes stained for DAPI (blue staining), GFAP (red staining), beta III tubulin (green staining) and TUNEL (white staining). Arrows denote apoptotic cells. (E) Quantification of apoptosis in mixed cultures of neurons and astrocytes, 16–17/18-20 wpc (data was combined due to not significant comparing both). Clearly, neurons in these cultures were highly susceptible to apoptosis (red line, (*p≤0.034 compared to Mock, n=3 independent donors in triplicate); however, astrocytes in the culture were negative for TUNEL, as indicated in B. No significant apoptosis was detected in the ZIKV-infected cultures compared to mock infection. (F) Quantification of apoptosis using mixed cultures of neurons and astrocytes, 21–22 wpc. No neuronal apoptosis was detected (red line), astrocyte apoptosis was negative, and C indicated a high dependency on the culture’s development or time of isolation. No significant apoptosis was detected in the ZIKV-infected cultures compared to mock infection (*p≤0.034 compared to Mock, n=3 independent donors in triplicate).

To determine the consequences of ZIKV infection in neurons, we infected pure cultures of astrocytes or mixed cultures of neurons and astrocytes using cultures from different developmental ages, as indicated in the previous figures. We evaluated apoptosis using staining for nuclei (DAPI, blue staining), glial marker (GFAP, red staining), beta III tubulin (green staining), and TUNEL (white staining). Analysis of pure cultures of astrocytes indicated that ZIKV infection (MOI, 0.1/1) did not induce significant apoptosis at any of the cultures obtained at 16–17, 18–20, and 21–22 wpc (Figure 5A, representative confocal image). Quantification of the apoptotic levels in pure cultures of astrocytes (16–17/18–20 wpc) indicates apoptosis is not detected despite the alterations in inter-organelle interactions and viral accumulation (Figure 5B). Also, no significant changes in the total number of astrocytes was detected indicating that ZIKV infection is not lytic (data not shown). Analysis of astrocyte cultures obtained at 21–22 wpc also did not apoptose (Figure 5C).

In contrast, ZIKV infection of mixed cultures of neurons and astrocytes indicated significant neuronal apoptosis, beta-tubulin III positive cells (Figure 5D, representative confocal image). Quantification of apoptosis in the mixed cultures indicates that ZIKV infection did not compromise astrocyte apoptosis but increased neuronal apoptosis in a time-dependent and neurodevelopmental manner according to the isolation time of the cells (Figure 5E, 16–17/18–20 wpc, *p≤0.034 compared to Mock, n=3 independent donors in triplicate). However, ZIKV infection of the mixed cultures obtained at the developmental stages, 21–22 wpc, did not result in significant neuronal or astrocyte apoptosis (Figure 5F), supporting that ZIKV neuronal toxicity has a specific developmental window to induce CNS dysfunction as indicated in Figure 1. Overall, our data indicate that ZIKV infection of astrocytes is extensive and accumulate a large amount of virus within enlarged vesicles in conjunction with interorganelle interaction dysfunction. ZIKV infection resulted in the apoptosis of neurons in a developmental-dependent manner.

## Discussion

ZIKV has been well-established to compromise brain development if the mother becomes infected with the virus and it is transmitted to the fetus. The more evident brain compromise associated with ZIKV is microcephaly, or other neurodevelopmental issues in the fetus, including decreased brain volume, apoptosis, inflammation, and disorganization of neuronal layers of the cortex [6, 26, 27, 43, 59]. However, ZIKV induces apoptosis, pyroptosis, and autophagy, compromising CNS development. Also, ZIKV infection has a particular neurodevelopmental window that requires further investigation.

ZIKV is a single-stranded RNA virus that belongs to the *Flaviviridae* family and genus Flavivirus genus. The emergence of other viruses, such as SARS-CoV-2, “hides” the active status of this virus in several areas of the world. But the long-term consequences of ZIKV infection in the mother and fetus are still active and ongoing. Currently, several Flaviviruses are neurotropic such as dengue viruses, West Nile, and yellow fever viruses, but also emerging viruses, such as Japanese encephalitic, tick-borne encephalitis and Usutu viruses [68], [69], [70], [71]. Despite extensive investigation and vaccines (experimental and under ongoing use), these viruses still pose a serious public health challenge and concern due to the infection and the expansion of the vectors worldwide [71].

Overall, the clinical symptoms of acute flavivirus infection range from mild to severe life-threatening diseases, including hemorrhagic fever, shock, encephalitis, paralysis, hepatic failure, and congenital defects. Several groups determine that severe disease is correlated with polymorphisms in critical host genes, including CCR5 for West Nile, DC-SIGN for Dengue, age, comorbidities, immune status, and prior flavivirus immunity [70, 72], [73], [74]. In the case of ZIKV, direct and indirect neuronal and neuroprogenitor cell damage has been described [6, 11, 12, 25, 26, 29, 59]. Analysis of brain fetuses infected with ZIKV indicates two specific mechanisms of damage: first, neuronal cell death, glial/microglia activation, and infiltration of monocytes, CD4^+^ and CD8^+^ T lymphocytes and second, exacerbated inflammation [13, 55, 59].

Although clinical diagnostic measures and public awareness were greatly increased after the world health organization identified ZIKV as a Public Health Emergency of International Concern from February 2016–May 2017, the mechanism of viral infection within the developing brain remains largely unknown. This is partly due to the timeline of maternal ZIKV infection, breach of the placental barrier and the resulting developmental problems in the fetus can be traced as 6 wpc until as late as 29 wpc [75], but the main consensus is indicated in Figure 1. Also, despite the evolution of several animal models, most data are limited to ultrasound, postmortem tissues, and newborn information, however, the specific developmental windows and consequences in the fetus development still are not well understood [6, 9, 59, 70, 73].

Our data using primary ZIKV isolates, no animal adapted, indicates that ZIKV infection of human astrocytes or mixed cultures of neurons and astrocytes had profound results in a viral release, infectivity, and viral protein production, but more important, in neuronal apoptosis. Our data indicate that the infection of astrocytes and mixed cultures depended on the wpc. The early stages of development, 16–17 and 18–20 wpc supported high infection and neuronal apoptosis. However, astrocytes and mixed cultures obtained at 21–22 wpc were less sensitive to ZIKV infection, viral production, organelle interaction compromise, accumulation of ZIKV in enlarged vesicles, and associated neuronal apoptosis. Our confocal and TEM data underscore the high astrocyte infectivity in a developmental-dependent manner with a significant longer viral presence due to the high accumulation of virions inside vesicles of infected astrocytes that last for extended periods, providing an alternative explanation by which ZIKV, even in the absence of viral replication, still could induce neurodevelopmental and neurologic compromise even if the active infection is not detected. Thus, astrocytes are the main target for ZIKV infection and associated neurological damage, but they also play a key role as a viral reservoir. Our work complements previous work that astrocytes are targeted by Zika and other flaviviruses [76], [77], [78], [79], [80], [81], but for the first time, we are demonstrating that astrocytes are highly sensitive and can maintain massive accumulation of virions internally in a developmental-dependent manner. Thus, astrocytes must be considered a key player in ZIKV neurodevelopmental complications as well as a key ZIKV reservoir.

Astrocytes, in general, are poorly considered in several brain-related diseases; most groups consider astrocytes as a cell type that responds to injury-inducing inflammation and scarring; however, this concept of astrocytes being just an observed is changing rapidly. For example, in NeuroHIV, astrocytes were not considered until recently. For decades astrocytes’ HIV infection was considered minimal to undetectable, residual, and unproductive. However, our work demonstrated that despite minimal entry, replication, and cell death, few HIV-infected astrocytes could have devastating consequences in the HIV-infected population despite undetectable replication [82], [83], [84], [85], [86]. We demonstrated that HIV-infected astrocytes, like ZIKV-infected astrocytes, are a reservoir for the virus and become protected from apoptosis despite significant alterations in most organelles and their interactions [82, 83]. Minimal residual HIV replication or long-lasting viral protein production, like in ZIKV infection, generated bystander damage into neighboring uninfected cells, including neurons, endothelial cells, and other uninfected astrocytes [83, 85], [86], [87], [88], [89], [90]. In agreement, ZIKV infection of astrocytes compromised bystander CNS damage by surviving the infection, compromising signaling and inter-organelle interactions, and synaptic compromise. We must denote that astrocyte ZIKV infection is not only permissible but also efficient since infectious virions are secreted within 24 h post-infection and stored in high numbers in vesicles, probably to generate long-term damage even in the absence of new viral replication.

Others have identified neuronal cells and neuronal precursor cells as major targets for ZIKV infection – cell types that are predominant in the developing brain until 17 wpc and present a strong mechanism of infection during the early stages of development. Early maternal infection during gestation is identified by severe microcephaly and fetal demise and miscarriage [75]. *In vitro* studies using tissue from 9–13 wpc resulted in glial infection and reduced neuronal proliferation [47]. Human and chimpanzee iPSC clones differentiated into neurons and neurospheres underwent apoptosis after inoculation with ZIKV, and *in vivo* murine models demonstrated NPCs are significant targets and decreased proliferation that results in a microcephaly phenotype in pups [91]. Examination of total cellular tropism *in vitro* using slide culture of the human fetal brain from 9 wpc recognized that although neuronal cells and NPCs are infected, glial cells are infected and result in clustering due to the permissibility of ZIKV through the cell surface receptor AXL [50]. Thus, our data and the existing data support that glial cells are major cellular targets for ZIKV infection, and further studies are needed to understand their role in ZIKV pathogenesis.

We propose that ZIKV-infected astrocytes compromise the cell migration of the neurons into the appropriate position in the brain. Astrocytes are critical for the proper destiny and formation of future brain structures [92], [93], [94], [95], [96], [97]. Several diseases and animal models indicate that impaired neural cell migration leads to malformations, as described in ZIKV infection, including autism, schizophrenia, and other developmental brain issues. Our data support that infection for 16–22 wpc astrocytes is highly dysregulated, and future studies will explore their nursing capabilities and their role in synaptic stability, axonal guidance, and neuronal migration. An alternative explanation is that infected ZIKV astrocytes compromise the differentiation and self-renewal capabilities of astro/neuronal progenitor cells, mainly controlled by differentiation factors that probably are altered by ZIKV infection. All critical points are described in ZIKV-associated abnormalities.

A critical point that needs to be addressed is how the immune or genetic status of the host contributes to the disease. There is significant controversy about whether cross-reactive anti-DENV antibodies can enhance ZIKV infection and pathogenesis [98], [99], [100], [101], [102], [103], [104]. In a macaque study, ZIKV infection enhanced DENV viremia and delayed immune response [105], [106], [107], [108], [109]. These inter-viral interactions could have devastating consequences if vaccines cross-react, further enhancing the pathogenesis of other flaviviruses. Currently, the answer is still uncertain and open highly controversial approaches that could result in higher pathogenesis and require aggressive investigation to prevent unwanted consequences by cross-reactivity among different flaviviruses.

In conclusion, astrocytes are ZIKV reservoirs with the unique characteristic of infectivity (developmental dependent), protected from apoptosis, become major stores for ZIKV, and participate in bystander neuronal damage. Our data indicate that ZIKV in astrocytes need to be considered a key cell type to prevent or reduce the onset of neurodevelopmental consequences of ZIKV in the fetal brain and to help design new treatment to reduce bystander damage.

## Materials and methods

### Materials

DMEM, MEM, Neurobasal media, N2 supplement, penicillin/streptomycin, trypsin-EDTA, DNAse, HEPES solution, sodium bicarbonate, non-essential amino acid solution, GFAP antibody, and Prolong Anti-fade with DAPI were all purchased from Thermo Fisher Scientific (Waltham, MA, USA). Fetal bovine serum was purchased from Atlanta biologicals (Lawrenceville, GA, USA). Zika NS1 rabbit polyclonal antibody (GTX133306) was purchased from Genetex (San Antonio, TX, USA). Zika Virus strain PA259459 was obtained from the World Reference Center for Emerging Viruses and Arboviruses through the University of Texas Medical Branch (UTMB) at Galveston (Galveston, TX, USA). All other materials were purchased from Sigma-Aldrich (St. Louis, MO, USA).

### Source of human fetal tissue.

Fetal tissues were obtained after elective abortions from healthy females and procured by Advanced Bioscience Resources, Inc (Alameda, CA, USA). The tissue collection protocols were approved by Rutgers University and the University of Texas Medical Branch Institutional Review Board (Protocol Numbers, Pro20140000794, Pro2012001303, 18–0136, 18–0135, 18–0134).

### Tissue collection and preparation of human astrocyte monolayers

Tissue preparation was prepared from previous literature, with some changes [110]. Human fetal CNS tissue was cleared of meninges using tweezers and then mechanically dissociated into a slurry. The tissue slurry was then evenly distributed into as many as six by 50 mL conical tubes and brought to 40 mL total volume per tube using HBSS supplemented with 2% penicillin/streptomycin solution. Next, 4 mL of 2.5% Trypsin-EDTA and 180 µL of DNase I Solution (>2500 U/mL) were added to each conical tube. Tubes were secured and rotated at 37 °C for 45 min. Following rotation, 3 mL FBS is added to each tube to inactivate Trypsin-EDTA. Next, solutions are filtered using the following setup: Assemble two separate 2-piece Bxchner funnels containing a 250 µm nylon mesh filter or a 150 µm nylon mesh filter. Place funnels over the new 50 mL conical tube and filter tissue-containing solution through the 250 µm filter. Take flow-through tissue solution and filter through the funnel containing the 150 µm filter into a clean 50 mL conical tube. Repeat for each 50 mL conical tube containing tissue solution. To increase yield, use plastic Bchner funnels and remove the porous surface of the plastic before use so that only the nylon filters impede flow-through.

Centrifuge tissue contains a solution in a swinging bucket rotor for 10 min at 300 × g and room temperature. Remove supernatant with care not to disturb the pellet. Resuspend pellet in HBSS containing 2% penicillin/streptomycin and centrifuge in a swinging rotor bucket again for 10 min at 300 × g and room temperature. Remove supernatant and replace with growth medium. The growth medium comprises DMEM supplemented with 10% FBS, 2% Penicillin/Streptomycin, 2% non-essential amino acids and 25 mM HEPES solution at pH 7.4. Seed 125 cm^2^ flasks with a cell density of 10^5^–10^7^ cells per flask. Incubate at 37 °C and 5% CO_2_ for 12 days. After 12 days, microglia cells are removed by shaking, as described previously [111]. Passage cells at a 1:3 split ratio. After three passages, neurons will die, and cultures will be >95% GFAP + astrocyte cells.

### Virus propagation and concentration.

Cell-free virus stocks were obtained from the World Reference Center for Emerging Viruses and Arboviruses through UTMB. Lyophilized virus was resuspended in 1 mL of DMEM containing 2% Penicillin/Streptomycin (5000 units/mL and 5000 μg/mL concentration) prior to inoculation of 75 cm^2^ flasks containing confluent C636 mosquito cells. A total of 10 mL of medium without serum was added, and flasks were incubated at 29 °C and 5% CO_2_ for 2 h with gentle rotation every fifteen minutes to prevent drying of the monolayers. After 2 h, media was removed and added to an Amicon^®^ Ultra – 15 centrifugal filters with NMWL 100,000 and a maximum volume of 15 mL per tube. Centrifugal filters were loaded into a swinging bucket rotor and centrifuged for 20 min at 4000 × g at 4 °C. Concentrate was recovered from the inner filtrate device, aliquoted, and stored at −80 °C until future quantification or use.

### Plaque quantification of cell supernatants.

In preparation for stock or cell supernatant viral quantification, Vero cells were plated onto six-well plates at a density of 5e^5^ cells/well in 5 mL of media per well. This seeding density results in approximately 85% confluency within 24 h.

On the day of the assay, ten-fold (viral stocks) or two-fold (cell supernatant) dilutions are made using PBS. Serial dilutions have been completed five times with a total volume >1 mL per dilution. Next, 500 µL per dilution is added per well to administer a total of five different dilutions to the six-well plate. Supernatant from uninfected C636 cells (“mock control”), after clarification for 10 min at 2000 × g, is administered to the sixth well. Plates are then incubated at 37 °C and 5% CO_2_ for 1 h with rotation every fifteen minutes to prevent drying of the monolayer. Inoculum is removed from wells, and 4 mL of overlay solution is added per well. Overlay solution is composed of 50% of 1.2% (w/v) carboxymethylcellulose (CMC) in PBS, 46% 2X MEM media (without phenol red), 2% Penicillin/streptomycin, and 2% FBS. The resulting overlay media contains 0.8% CMC in supplemented 1X MEM (without phenol red).

Plates are incubated at 37 °C and 5% CO_2_ for 7 days. After 7 days, overlay solution is poured off the plates and monolayers are washed gently with PBS. Monolayers were fixed with 4% paraformaldehyde for 20 min, washed gently with PBS, and stained with 0.25% (w/v) crystal violet solution made in 30% ethanol in PBS. Visualization of plaques can be enhanced using a lightbox as needed.

Plaque forming units per mL (pfu/mL) are calculated using the following formula:

$$\text{Concentration}\frac{pfu}{mL}=\frac{\left(\text{Number of plaques}\right)}{\text{Dilution factor }x\text{Volum}{\text{e}}_{\text{inoculum}}}.$$

### Immunocytochemistry

Cells were plated onto a glass coverslip before fixation with 70% ice-cold ethanol for 30 min on ice. After fixation, coverslips were washed with ice-cold PBS and blocked with blocking solution overnight at 4 °C or for 1 h at room temperature. Blocking solution comprises: PBS supplemented with 50 µM EDTA, 0.9% fish gelatin, 1% horse serum, and 5% (w/v) bovine serum albumin. After blocking, rabbit anti-Zika NS1 peptide antibody (Genetex) and mouse anti-GFAP were incubated at dilutions of 1:1000 in block solution overnight at 4 °C in a humidified incubation chamber. Samples were brought to room temperature and washed with PBS five times before incubation with anti-mouse Alexa Fluor 647 and anti-rabbit Alexa Fluor 488 for 2 h at room temperature in a humidified incubation chamber. Before the acquisition, coverslips were washed five times with PBS and loaded onto glass slides using Prolong Gold Anti-fade reagent with DAPI (Thermo Fisher) and incubated at room temperature in the dark overnight. The acquisition was completed on a Nikon A1+ Confocal Microscopy, and images were analyzed using Nikon NIS software.

### Transmission electron microscopy (TEM)

The Albert Einstein College of Medicine Analytical Imaging Facility completed transmission electron microscopy preparation and acquisition. Briefly, cell monolayers were grown on 60 mm dishes and washed with serum-free media for 1 min prior to fixation using 2% paraformaldehyde + 2.5% glutaraldehyde in 0.1 M Cacodylate buffer at room temperature for 30 min or overnight at 4 °C. After fixation, monolayers were rinsed with PBS and dehydrated with a series of ethanols before the addition of propylene oxide and embedment in liquid epoxy resin. Sections of 80 nm thickness were collected using ultramicrotomy and stained with uranyl acetate and lead citrate before imaging on a JEOL 1200EX transmission electron microscope at 80 kV. Images were then collected with a Gatan Orius camera using Digital Micrograph.

### Cell culture

All cells, except C636, were maintained at 37 °C and 5% CO_2_. C636 cells were maintained at 28 °C and 5% CO_2_ for optimal growth. Human fetal astrocytes were maintained in DMEM supplemented with 10% FBS, 2% penicillin/streptomycin, 2% non-essential amino acids, and 25 mM HEPES solution. C636 cells were maintained in RPMI media supplemented with 5 mL of 5% (w/v) sodium bicarbonate, 1% penicillin/streptomycin, 10% FBS, 1 mM sodium pyruvate, and 1% nonessential amino acids. Vero cells were maintained in DMEM supplemented with 10% FBS and 2% penicillin/streptomycin.

### Image analysis

Raw data for confocal analysis and 3D deconvolutions were obtained using NIS elements (Nikon, Japan). The colocalization, intensities, lengths, and stability were quantified in NIS elements and Image J.

### Statistical analysis

Data were analyzed using Origin 8.1 (Northampton, MA, US). For single comparisons, Student’s *t*-test was performed. For multiple comparisons, mean differences were tested by non-parametric Kruskal–Wallis analysis and adjusted using the Bonferroni–Dunn correction. p values of < 0.05 were considered significant.
